# 

**Published:** 1918-03-30

**Authors:** 


Rates of Subscription: Twelve months, 6/6; Six months, 4/-; three months, 2/-, post free to any address in the United Kingdom. Abroad, Twelve months, 8/8; Six months, 5/-; Three months, 2/6, post free. **THE HOSPITAL "Index" to Volume LXII, April 1917 to September 1917, is now ready, price 2½d. per copy, post free.** Address The Manager, The Hospital, "The Hospital" Building, 28/29 Southampton Street, Strand, London, W.C. 2.

### Some Annual Meetings.

### THE ITALIAN HOSPITAL.

At the annual general meeting of the governors of the Italian Hospital, held last week at Queen Square, W.C., both Lord Edmund Talbot, M.P., and Sir Dyce Duckworth, Bart., M.D., expressed the opinion that the work and benefits of the hospital were not adequately recognised. Last year it cared for 530 military in-patients—484 British and 46 Canadian. Of the 392 civil in-patients 225 were Italian, 129 British, and 38 of other nationalities; while out of a total of 2,733 out-patients 1,746 were Italian, 778 British, and 209 of other nationalities. Of the total number of patients treated, therefore, more than one-third were British. During the year under review the hospital had been approached by the War Office with a view to providing further beds and accommodation for soldiers, but upon investigation the authorities were of opinion that the institution was doing all it possibly could in that direction.

### MOUNT VERNON HOSPITAL.

**Several speakers at a meeting of the Mount Vernon Hospital for Consumption and Diseases of the Chest, held last week at Fitzroy Square, W., referred to the great loss the institution had sustained by the death of Mr. Charles Johnston, who had been chairman of the committee of management since 1911, and of Dr. J. Edward Squire, C.B., the consulting physician, whose association with the hospital dated from 1881. Mr. J. S. Fletcher, M.P., who presided, viewed with dismay any State control of hospitals and their transfer to "salaried officials." He pointed out that our national character had been altered by the war, which had brought about the loss of personal independence and liberty, inasmuch as everyone was now under the orders of the State.**

### LONDON LOCK HOSPITAL AND RESCUE HOME.

**"We have been approved by the Local Government Board, and now receive a 'grant in aid.'" Those encouraging words were addressed to the governors of the London Lock Hospital and Rescue Home by Mr. J. F. W. Deacon, deputy-chairman of the board of management, who presided, in the absence of Lord Kinnaird, at a meeting held last week at Dean Street, Soho. But, despite the grant, he pointed out that at least £9,000 was needed annually to maintain the institution, which last year dealt with 40,000 cases, coming from many counties in England and Wales, from Ireland, Scotland, the Colonies, and the countries of the Allies.**

### Matrons' and Sisters' Appointments.

CHISWICK HOSPITAL.—Miss Frances Greenland has been appointed sister. She was trained at the Huddersfield Royal Infirmary, and has been sister at Clayton Court Hospital, East Liss.

DR. BARNARDO'S HOME FOR CRIPPLED CHILDREN, BIRKDALE.—Miss Elsie Key has been appointed sister. She was trained at the Royal Infirmary, Lancaster, and has since done private nursing.

GRANGE HOSPITAL, SOUTHPORT.—Miss Florence Jones has been appointed ward sister. She was trained at the Highfield Infirmary, Liverpool, and has been ward and theatre sister at the 3rd Western General Hospital, Newport, Mon.

MANSFIELD AND DISTRICT HOSPITAL.—Miss R. Farrell has been appointed sister. She was trained at Lambeth Infirmary, and has been doing private nursing.

NORTH LONSDALE HOSPITAL, BARROW-IN-FURNESS.—Miss H. E. Macdonald has been appointed night sister. She was trained at the above hospital, and has since been nurse-in-charge of the x-ray and V.D. departments there.

ST. JAMES' INFIRMARY, BALHAM.—Miss Bertha Thornhill has been appointed ward sister. She was trained at Wolverhampton Infirmary, where she was later staff nurse, and holds the certificate of the Central Midwives Board. She has also been sister of Shadlow Infirmary, day and night sister at Coventry Infirmary, and sister at Manchester Poor-Law Infirmary. Miss Muriel Davis has been appointed home sister. She was trained at Charing Cross Hospital, afterwards being ward sister there.

STOCKTON AND THORNABY HOSPITAL, STOCKTON-ON-TEES.—Miss Florence Reeves has been appointed sister. She was trained at Leicester Royal Infirmary.

UNION INFIRMARY, PLYMOUTH.—Miss S. A. Lewis has been appointed home and theatre sister. She was trained at Crumpsall Infirmary, Manchester, and has since been charge nurse at Plymouth and Wolverhampton Union Infirmaries, superintendent nurse of Barnsley, Congleton and Bishop Stortford Union Infirmaries, night superintendent at Brighton Union Infirmary, and ward sister at the Military Hospital, East Preston.

WEST CORNWALL DISPENSARY AND INFIRMARY, PENZANCE.—Miss Helen M. Lapham has been appointed matron. She was trained at the Bradford Royal Infirmary, has been sister at the Royal Eye and Ear Hospital, Bradford, and ward sister, night superintendent, and holiday assistant matron at the York County Hospital.

WILDERNESSE MILITARY HOSPITAL, SEVENOAKS.—Miss Amy Phipps has been appointed matron. She was trained at St. George's Infirmary, London, where she was later charge sister. She has been night superintendent at Wildernesse Hospital and theatre sister at the Emergency War Hospital, Arc-en-Barrois, France.

---

**NOTE.—Particulars of Appointments for insertion in this column will be welcomed.**

☛ For Nursing Affairs in Ireland, by "Ierne," see p. 554; Mr. G. Q. Roberts on St. Thomas' Hospital History, see p. 560.

